# Young Women’s Experiences With Technology-Facilitated Sexual Violence From Male Strangers

**DOI:** 10.1177/08862605211030018

**Published:** 2021-07-16

**Authors:** Alisha C. Salerno-Ferraro, Caroline Erentzen, Regina A. Schuller

**Affiliations:** 1 York University, Toronto, ON, Canada; 2 University of Toronto, ON, Canada

**Keywords:** sexual harassment, stalking, adult victims, sexual assault

## Abstract

Stranger-perpetrated harassment was identified decades ago to describe the pervasive, unwanted sexual attention women experience in public spaces. This form of harassment, which has evolved in the modern era, targets women as they navigate online spaces, social media, texting, and online gaming. The present research explored university-aged women’s experiences (n = 381) with online male-perpetrated sexual harassment, including the nature and frequency of the harassment, how women responded to the harassment, and how men reportedly reacted to women’s strategies. Trends in harassment experiences are explored descriptively and with thematic analysis. Most women reported receiving sexually inappropriate messages (84%, n = 318), sexist remarks or comments (74%, n = 281), seductive behavior or come-ons (70%, n = 265), or unwanted sexual attention (64%, n = 245) in an online platform, social media account, email, or text message. This sexual attention from unknown males often began at a very young age (12-14 years). The harassment took many forms, including inappropriate sexual comments on social media posts, explicit photos of male genitalia, and solicitations for sex. Although most women reported strong negative emotional reactions to the harassment (disgust, fear, anger), they generally adopted non-confrontational strategies to deal with the harassment, electing to ignore/delete the content or blocking the offender. Women reported that some men nevertheless persisted with the harassment, following them across multiple sites online, escalating in intensity and severity, and leading some women to delete their own social media accounts. These results suggest the need for early intervention and education programs and industry response.

## Introduction

Marie decides to unwind at the end of a long workday by scrolling through her Facebook account. A man she does not know, a friend of a friend, has posted an offensive sexual comment in response to her recent post about politics. She cringes, as her family, friends, and some coworkers see her Facebook posts. She deletes her post in embarrassment. Angela is a university student who spends hours each day on her phone texting and using social media. She receives a text message from a male she does not know; the message contains a picture of a man’s genitalia with a request for her to send the same. She deletes the picture but remains shaken by the graphic invasion.

These experiences are part of a growing phenomenon of technology-facilitated harassment of women by men who are either strangers to them or barely acquaintances. Little is known about the prevalence of online sexual harassment, the forms it might take, how it impacts women’s lives, or the strategies women engage in to deal with this unwanted male attention online. The present research bridges the literature on in person stranger harassment to that which is facilitated by technology in an online world. We begin with a review of previous research exploring women’s experiences with stranger harassment, followed by a discussion of the emerging research on technology-facilitated sexual violence (TFSV; Henry & Powell, 2018). Finally, we turn to the present study where we explore the frequency of online sexual harassment of women, the forms it often takes, and its consequences.

## Stranger Harassment (Traditional Forms)

Fairchild and Rudman (2008) define stranger harassment as receiving unwanted attention from a stranger that is sexual in nature, which can include physical, verbal, and nonverbal harassment. Two key concepts separate stranger harassment from nonstranger harassment, with the former occurring in public places and involving a perpetrator that is unknown to the victim (Macmillan et al., 2000). Stranger harassment is sometimes referred to as street harassment, referencing the fact that stranger harassment traditionally occurred while women were out of home or in the “streets” (Vera-Gray, 2016). This includes the literal streets, but also other public places such as bars, clubs, and stores or shopping malls.

Macmillan et al. (2000) were some of the first researchers to empirically investigate stranger harassment, drawing on items taken from the national Violence Against Women Scale (VAWS) that was originally administered in 1993 (Johnson & Sacco, 1995). The researchers found that 85% of women reported experiencing some form of stranger harassment, finding that women were more likely to experience stranger harassment than nonstranger harassment, which approximately 51% of respondents reported. Unfortunately, these prevalence rates have not abated over the 20 years since the VAWS was first administered. A landmark study in stranger harassment conducted by Fairchild and Rudman (2008) found that 40% of college women experienced stranger harassment once a month, while 31% experienced stranger harassment every few days. A quarter of their respondents reported experiences of stranger harassment tantamount to sexual assault, like forceful grabbing or fondling, and unwanted touching or stroking.

Thompson and Cracco (2008) conducted a survey of college men across the United States, assessing whether they had engaged in a variety of consensual and nonconsensual sexual acts with both acquaintances and strangers. In their study, two-thirds of respondents admitted to pressing up against a woman’s body from behind without her consent, and 80% had grabbed a woman’s backside at a party without her consent. More than half reported they had rubbed or stroked the knee of a woman they didn’t know well, and a third asked a woman they didn’t know to have sex. The men viewed these behaviors as normal, reflecting a well-established and societally constructed belief that men are entitled to sexual contact with women (Cairns, 1994; Mann & Beech, 2003). This sexual entitlement plays into larger dynamics of gendered power relationships, in which women are sexualized objects to which men have presumptive access (Cairns, 1994). While some men may mistakenly believe that their actions will be well-received by women, many men engage in these practices for their own gratification, amusement, or status with other males (Gailey & Prohaska, 2007). Bailey (2017, p. 599) notes that stranger harassment imposes unwanted “heterosexual intimacy” on women, reflecting the presumed right of men to dominate women in public spaces. Women have reported that this behavior, although unwanted and intimidating, is something that they simply must tolerate if they want to go out (Brooks, 2011).

The negative consequences of street-level stranger harassment on women are well documented and include feelings of personal vulnerability (Bastomski & Smith, 2017), increased fear of being raped (Fairchild & Rudman, 2008), and a heightened belief in the likelihood that they will be the victim of gender-based violence (i.e., rape or intimate partner violence: Donnelly & Calogero, 2017). Del-Greco and Christensen (2019) observed that street-level harassment was positively correlated with anxiety, depression, and disrupted sleep, suggesting that the harassment is anything but harmless. These issues become compounded when we contemplate the changes in public spaces, particularly as we become more connected online.

### Stranger Harassment in an Online World

Given the rapid advancements in digital technology and the ever-increasing popularity of social media and smart phones, the scope of “public” has evolved since stranger harassment was first explored in the 1980s. Although in the past, strangers were most often encountered in physical public spaces (streets, night clubs, stores), the internet has now become a commonly frequented space in which one may encounter strangers on a regular basis. Some websites and mobile phone apps, online gaming, and social networking platforms (e.g., Facebook, Twitter, Instagram), actively facilitate and encourage connections between strangers. While these online spaces create environments in which to socialize and engage with new people, they can also provide opportunities for unwanted sexual harassment and attention. Indeed, given the anonymity and deindividuation provided in these forums, the online world is an ideal ground for predatory behavior. The present research is concerned with unsolicited, nonconsensual online communication from men who are unknown to their female recipients. In the present paper, we use the broader term TFSV (Henry & Powell, 2018), as it encompasses a wide variety of sexually harassing behaviors that may be committed by strangers. Henry and Powell (2018, p. 195) define TFSV as “a range of criminal, civil, or otherwise harmful sexually aggressive and harassing behaviors that are perpetrated with the aid or use of communication technology.” TFSV, they argue, can take many different forms including harassing speech that involves unwanted sexual attention, causes fear or apprehension in the victim’s mind, solicits sexual activity, or disseminates unwanted explicit sexual photos, among other things. TFSV can occur on social media, dating websites, via texting, or even through Bluetooth technologies such as AirDrop. While noting that research in this area is “extremely sparse,” their literature review of available research indicated that younger women received the disproportionate share of this form of harassment.

Cuenca-Piqueras et al. (2019) note that stranger-perpetrated TFSV markedly differs from street-level harassment. In an online context, the aggressor is sometimes anonymous and able to harass without repercussion; as a result, online sexual harassment may involve stronger and more offensive language. There is little recourse for women who experience online forms of stranger perpetrated TFSV, as the offending post may be deleted or made anonymously. Even where the identity of the harasser is known, there are limited remedies available. Men who have been blocked from contacting women online can easily create new accounts, obtain new user profiles, or engage in stalking behavior. As an emerging field, there is limited research exploring the psychological impact of online sexual harassment on women or what resources they have available to deal with such harassment. In the context of online gaming, Cote (2017) found that female gamers actively managed their online gaming environment to avoid male sexual communication, taking measures to avoid revealing their gender, adopting aggressive personality traits to ward off potential predators, or maintaining a high level of skill and experience. Similarly, Megarry (2014) conducted a case study of the #mencallmethings hashtag, in which women shared their experiences of verbal harassment, largely through technology-facilitated media. Megarry’s analysis of these accounts showed that the abuse women regularly receive online impedes their freedom of expression.

One new form of TFSV has emerged in which men share unsolicited photos of their genitalia with women, known colloquially as “dick pics,” via text, email or messaging applications (Hayes & Dragiewicz, 2018). In a recent study, Mandau (2020) interviewed 29 young adults (both men and women) about their experiences sending and receiving unsolicited “dick pics.” In that study, women generally experienced the photos as intrusive and incompetent attempts at flirtation, whereas the men believed that it was a normalized way to show off, compliment women, or seek sexual reciprocation. Similarly, Oswald et al. (2019) found that approximately half of the men in their study sent “dick pics” in the hope of reciprocation, one-third were trying to obtain a sexual partner and 18% experienced sexual arousal by sending the photos. A minority had overtly harmful motives, hoping the recipient would feel shock (17%), fear (15%), anger (11%), disgust (9%), shame (8%), and devalued (6%). The authors noted that some men may be playing a game of numbers, hoping that the more “dick pics” they send, the more likely it is that at least one woman will respond positively (Oswald et al., 2019; Segran & Truong, 2016).

The active process of sending unsolicited nudes to a stranger is not an innocuous or benign activity. Although displaying one’s nude genitalia to unknown women in person would constitute a criminal offence and possibly be diagnosed as a symptom of a mental illness (i.e., exhibitionism: Bader et al., 2008), distributing unsolicited “dick pics” to unknown women has become a much more widespread practice (Oswald et al., 2019; Waling & Pym, 2019). Waling and Pym (2019) studied the way “dick pics” are framed during discussions and online communities, noting a common cultural theme that heterosexual men lack insight or understanding into women’s sexual arousal or sexual needs (e.g., believing that visual imagery will lead to arousal for all women). The mere fact that the behavior occurs in an online space does not render it harmless, and it is comparable to obscene phone calls, flashing, and peeping activities, which regularly occurred in the 1980s (McGlynn et al., 2017).

Less is known about the effects of stranger-perpetrated TFSV on women, although there is a newly emerging literature. Receiving unsolicited photos of a man’s genitals is reported to be an almost universally aversive experience for women (Kohn, 2017; Waling & Pym, 2019), with women generally reporting unpleasant emotional reactions including shame, disgust, objectification, and anger (Vitis & Gilmour, 2017; Waling & Pym, 2019). Moreover, some women find the act coercive and feel that they are being manipulated into sending a similar picture in response (Ross et al., 2016).

### Present Research

The purpose of the present research was to explore women’s experiences of stranger-perpetrated TFSV in the 21st century, and the forms that it may take in an online space, specifically looking at the prevalence, frequency, and nature of harassment online. As well, the emotional impact that such harassment has on women, and the strategies they employ to deal with this unwanted online male attention was also explored. Although stranger-perpetrated harassment traditionally occurred on the streets and involved perpetrators completely unknown to the victim, stranger-perpetrated TFSV is sometimes perpetrated by individuals with a very remote connection to the victim (e.g., they may have an acquaintance in common, they may play the same online game). Very often, though, the perpetrators of TFSV are complete strangers to the victim. Thus, in the present study, we use a more broadly construed definition of the word stranger, to include both men who are unknown entirely to the victim as well as those who might have a tangential but almost nonexistent connection.

## Method

### Participants

Four hundred female participants were recruited from introductory psychology classes at a large Canadian university. Students were invited to participate in an online study for partial course credit. The study was advertised as a survey on women’s experiences with male harassment, though the current paper will only present the findings related to online sexual harassment. During the informed consent process, participants were advised that the study might address sensitive or triggering information pertaining to sexual harassment and that they could terminate the study at any time. Four participants failed to complete the study, and their data were removed from all analyses. Only those participants who self-identified as women were retained in the study. Four participants who identified as men and four did not disclose a gender identity and were removed from analyses. The final sample consisted of 388 participants, ranging in age from 18 to 38 years (*M* = 19.39, *SD* = 2.66). The sample was ethnically diverse, with 33.35% identifying as South Asian, 28.61% identifying as White, 10.82% identifying as Black, 10.05% as East Asian, 9.54% as Middle Eastern, and 7.74% identifying as a mixed or other heritage. There was also wide diversity in religious affiliation, with 40.98% identifying as Christian, 18.04% identifying as Muslim, 11.86% identifying as Hindu, 10.31% identifying as Atheist/Agnostic, 7.47% as Sikh, 6.19% as Jewish, 3.61% as Buddhist, and 1.55% as another religious affiliation or declining to respond.

### Materials

Prior to answering any questions, participants were provided with a definition of “stranger.” A liberal definition of the word was purposely used to capture “unfamiliar,” men, acknowledging that in the online world, many times women experience harassment via a friend of a friend, or someone they had only a brief interaction with online (e.g., on a dating app). For the purposes of the study, a stranger was defined as follows:

…someone whom you do not have a preexisting relationship with. This includes complete strangers (like people you would encounter on the street) AND people you may know, but do not necessarily have a personal relationship with, for example, a customer, coworker, a guy who lives on your street, or even a friend of a friend.

Participants were directed through a series of closed-ended and open-ended questions about their experiences with male stranger-perpetrated sexual harassment both online and in person. To capture as wide a range of experiences as possible, women were asked about a diverse array of male harassment. Six items were adapted from the VAWS (Fairchild & Rudman, 2008), and assessed whether participants had ever experienced, in an online context: (a) sexist remarks or behaviors, (b) seductive behavior, remarks, or come-ons, (c) crude and sexual remarks, jokes, or actions, (d) unwanted sexual attention or interaction, (e) subtle pressure/coercion to comply sexually, and (f) direct/explicit pressure/coercion to comply sexually. The remaining items from Fairchild and Rudman’s (2008) VAWS were not appropriate or applicable for online or technology-facilitated acts, as they primarily assessed physical contact or interpersonal interactions (e.g., unwanted touching, stroking, or hugging; forceful fondling or grabbing; whistles, catcalls).

To more fully explore the nature and frequency of technology-facilitated experiences, another four items were prepared a priori by the researchers. As there are no preexisting scales for TFSV perpetrated by strangers, the four items were generated based on experiences reported in prior studies exploring cyber harassment (Finn, 2004; Henry & Powell, 2016; Megarry, 2014; Oswald et al., 2019), which discussed common locations and vehicles for harassment (via text messages, social media, or gaming), as well as common forms of harassment experienced (pornographic images, “dick pics,” solicitation of sexual favors, and sexual or degrading comments). These additional items were meant to be exploratory in nature and included (a) inappropriate messages, (b) inappropriate pictures, (c) unsolicited nude pictures, and (d) offers of free gifts or money in exchange for sex. For each question, participants were asked to report whether they had experienced each type of harassment with a yes/no response.

If they responded in the affirmative, additional questions assessed how frequently they had experienced that form of harassment on an ordinal scale (1: a few times in my life, 2: a few times a year, 3: once a month, 4: twice a month, 5: every few days, or 6: multiple times per day), and on which platforms they had been received (e.g., Instagram, Facebook, Twitter). For each of these online locations, women provided frequency estimates, so that we could get an understanding of different patterns of online sexual harassment. Participants were also invited to provide an example of their experiences and were asked to describe the incident in as much detail as they were comfortable providing. This process occurred for each of the 10 questions assessing TFSV. We did not define “inappropriate,” instead leaving it up to respondents to decide what they considered was inappropriate. Based on the follow-up responses, respondents uniformly reported sexually inappropriate content (i.e., as opposed to other “inappropriate” content such as acts of cruelty, blasphemy, or other obscenity).

Participants were then invited to explain whether unwanted male sexual attention had changed their online behavior and were invited to explain in an open-ended manner how this might have changed their behavior. Finally, participants were asked to report the emotional reactions they experienced in response to the various forms of TFSV they encountered. Based on Russell’s (1980) circumplex model of affect, possible emotional reactions fell along two axes: valence (positive vs. negative) and physiological arousal (low vs. high). Thus, emotional reactions were either (a) positive or negative in valence, and (b) high or low in physiological arousal. Responses were provided on a 7-point scale ranging from 1: not at all to 7: very much, and included high arousal negative items (e.g., anger, fear), low arousal negative items (e.g., bored, sad), high-arousal positive items (e.g., excited, surprised), and low arousal positive items (e.g., calm, amused).

### Procedure

After providing informed consent, participants were invited to proceed through the survey questions at their own pace. The survey was administered through an online Qualtrics platform, and participants completed the survey outside of the lab to ensure confidentiality, anonymity, and reporting of sensitive information. Upon completion of all materials, participants were debriefed and thanked.

## Results

Closed-ended data were analyzed through frequency of responses and mean score endorsements were calculated. Open-ended responses were analyzed by the authors using a thematic analysis approach to understand what characterized their experiences beyond the created items. Thematic analysis is used to identify themes or patterns that emerge or repeatedly occur in the data. In accordance with Braun and Clarke’s (2006) guidelines, the authors read through and became familiar with the data, generating initial codes. We then analyzed and coded the data for these themes. Themes and codes were modified, assimilated and transformed throughout the coding process, as we moved from the initial codes to the final theme categories. In the final stages, themes were defined, and frequencies were tallied in accordance with traditional thematic analysis methods.

### Frequency and Nature of Stranger-Perpetrated TFSV

Table 1 provides an overview of how frequently women experienced various forms of TFSV. More than half of women reported receiving inappropriate messages (*n* = 207, 53.35%), approximately one-quarter received inappropriate pictures (*n* = 110, 28.35%), and unsolicited photos of male genitalia (*n* = 90, 23.30%). Many of the women provided descriptions of unpleasant experiences in which they received unwanted and unsolicited nude photos from complete strangers and without warning. For example, as one woman recounted “I went onto my Instagram and got a private message from an unknown person and I opened and it was a picture of a male’s genitals.” Ten percent of participants (*n* = 41, 10.57%) reported that they had received unsolicited offers of free items or gifts in exchange for sex. For example,

**Table 1.** Prevalence of Stranger-Perpetrated TFSV: New Items.

	Frequency
	A Few Times in My Life	A Few Times/Year	Once or Twice/Month^1^	Every Few Days	Multiple Times/Day	Total
Form of harassment						
Inappropriate messages	120 (30.93%)	43 (11.08%)	31 (7.99%)	11 (2.84%)	2 (0.52%)	207 (53.35%)
Inappropriate pictures	69 (17.78%)	23 (5.93%)	12 (3.09%)	4 (1.03%)	2 (0.52%)	110 (28.35%)
Unsolicited nude pictures	59 (15.21%)	14 (3.61%)	13 (3.35%)	3 (0.77%)	1 (0.26%)	90 (23.30%)
Offers of free stuff/gifts	30 (7.73%)	7 (1.80%)	2 (0.52%)	1 (0.26%	1 (0.26%)	41 (10.57%)

*Note.* Percentages correspond to percentage of the full sample of participants (*n* = 388).

^1^Responses of “once a month” and “twice a month” were collapsed to form a single category.

I had somebody ask me over Instagram if they could be my “pay pig.” When I asked him what that was he said he pays beautiful woman and then gave me his number because he was “ready to pay.” When I asked him what the catch was he said pictures in lingerie, phone calls, meet ups, etc. I told him no and he got quite angry.

Table 2 provides information about the nature and frequency of the TFSV women experienced across different platforms. Women reported experiencing it most commonly on Instagram, Facebook, and Snapchat. This most often took the form of sexist remarks or comments, inappropriate messages, seductive remarks, and come-ons. There were some variations with regards to which types of TFSV occurred in various platforms. Of the women who reported receiving inappropriate messages via technology (*n* = 207), the most common sources were Instagram (*n* = 107, 51.69%), Snapchat (*n* = 72, 34.78%), and Facebook (*n* = 52, 25.12%). A similar pattern was observed for inappropriate pictures: 104 women reported receiving such photos, most commonly received on Snapchat (*n* = 64, 59.81%) and Instagram (*n* = 38, 36.54%). Unsolicited nude photos (*n* = 90) were most commonly received on Snapchat (*n* = 58, 64.44%) and Instagram (*n* = 33, 36.67%).

**Table 2.** Prevalence of Online Harassment as a Function of Specific Site.

Original VAW items	Online Source						
	Instagram	Facebook	Twitter	Snapchat	Tinder	Dating Website	Total
Sexist remarks or behaviors	113	67	43	65	35	6	174
Seductive behavior, remarks, or come-ons	94	43	33	65	35	10	146
Crude and sexual remarks, jokes, or actions	45	22	21	34	20	9	77
Unwanted sexual attention or interaction	51	19	15	42	23	7	91
Subtle pressure/coercion to cooperate sexually	12	4	3	10	11	6	28
Direct/explicit pressure to cooperate sexually	6	2	1	6	4	1	12
Additional online items							
Inappropriate messages	107	52	29	72	28	7	207
Inappropriate pictures	38	25	10	64	9	6	110
Unsolicited nude pictures	33	21	8	58	7	6	90
Offered free gifts for sex	16	11	9	6	8	3	41

### Behavioral and Emotional Responses to Harassment

Women who experienced TFSV of any kind (*n* = 267) engaged in a range of response strategies, largely nonconfrontational in nature. As seen in Table 3, these strategies most commonly took the form of ignoring or deleting the offensive content or blocking the sender. Other, less common, strategies included asking him politely to stop, brushing him off politely, leaving the website, taking no action at all, and deleting one’s account. A small number of women reported that they played along with it, encouraged it, or tried to publicly shame the harasser. These nonconfrontational strategies should not be taken as evidence that the sexual harassment was innocuous. Looking specifically at the most frequent forms of harassment, and as shown in Table 4, exposure to online sexual harassment elicited negative emotional reactions from women. The most commonly reported emotional responses were high arousal negative emotions (e.g., disgust), and the least commonly reported emotional responses were high arousal positive emotions (e.g., excited). The lower arousal items fell between these two ends, with negative low arousal emotions more commonly endorsed than positive low arousal emotions.

**Table 3.** Women’s Responses to Online Harassment by Male Strangers.

	*N*	%^1^
Response to online harassment		
Ignored it	160	59.92
Blocked him	159	59.55
Deleted it	146	54.68
Told him politely to stop	49	18.35
Left the website/app	49	18.35
Tried to brush him off politely	40	15.00
Nothing	35	13.12
Deleted my account	22	8.24
Played along with it	22	8.24
Publicly shamed him	5	1.87
Encouraged it	5	1.87
Other	2	0.75

*Note.* ^1^Percentage reported pertains to the number of participants who reported experiencing online harassment (*N* = 267).

**Table 4.** Emotional Reaction to Male Stranger-Perpetrated TFSV.

	*M*	*SD*
Negative high arousal		
Annoyance	5.68	1.53
Disgust	5.19	1.82
Anger	4.46	2.01
Fear	4.11	1.88
Panic	3.75	2.04
Negative low arousal		
Confusion	3.35	1.95
Boredom	2.85	1.90
Sad	2.60	1.76
Shame	2.37	1.68
Positive low arousal		
Surprised	3.74	1.90
Calm	2.27	1.64
Amusement	2.05	1.55
Happy	1.61	1.05
Positive high arousal		
Sexy	1.77	1.27
Proud	1.52	1.02
Excited	1.48	0.97
Lust	1.45	1.03
Nothing/no reaction	2.60	0.86

## Exploring Stranger-Perpetrated TFSV Beyond the Scale Items

Of the respondents (*n* = 267) who reported that they had experienced stranger-perpetrated TFSV, 265 respondents opted to provide details regarding one or more of their experiences of being harassed by male strangers. Four main themes emerged, which are described below with exemplary quotes from respondents provided.

*Randomness.* A common theme that emerged from the open-ended responses (*n* = 40, 15.09%) involved the sheer randomness of the contact, particularly the randomness of the perpetrator’s identity. Participants described the messages as unexpected and out of context, with no natural progression leading up to the sexual messages. For example, the following participant described her experience as having “random strangers sending messages on snapchat or Facebook asking if I want to have sexual interactions with them.” One woman recalled a time that she posted a picture of herself to Instagram and received a direct message from a man she didn’t know “saying he liked me on my knees.” Similarly, “random males sent messages to me in my private messages that were very graphic (sexual), for example, they talk about having sex with me and getting me impregnated.” This random contact is perhaps unsurprising as we were assessing stranger-perpetrated behavior. It does, however, highlight the sense that women lack control over their online space and the ever-present threat of unwanted male intrusion.

*Happens all the time.* Some participants (*n* = 17, 6.42%) spoke about the sheer frequency in which they received harassment from strangers online, with a sense that it is normalized and frequent. For example, respondents used words like *constantly, usually, quite frequently,* and *happens all the time* to describe their experiences with stranger-perpetrated TFSV. For example, one participant described the following:

Almost every day on my social media account of unwanted nudity pictures by men. Every day or other day of nude pictures of penises. The men would want to send me chocolates, clothes and sometimes even marriage proposals. I would get sexual remarks based on my legs and body image.

These statements reflect the frequency and constancy of stranger-perpetrated TFSV that many women experience while simply existing and interacting in online spaces.

*Early age.* Respondents (*n* = 17, 6.42%) mentioned that their first experience with online male sexual harassment began at a young age, often between 12 and 14 years of age. For example, one woman recounted the following: “Back in the days of Blackberry Messenger I was more open talking to strangers because I was young…one time I just said ‘Hi’ and he replied ‘Hi’ then immediately after ‘Are you horny?’ I was only in elementary school.” Another woman reported that she “would get messages from an older man asking to take me out and he knew my age and that I was underage at the time.” Similarly, many respondents mentioned being asked about their virginity and sexual experience. For example, one woman recalled an experience that occurred during online gaming: “They have asked about my sexual nature (virgin or not) and more.” Another woman recalled that “a player online asked me if I knew how to have sex.” These questions about sexual inexperience suggest that the men were specifically targeting younger females, as it would seem unusual to ask this question of an adult woman.

*Persistence in the face of rejection.* Another common theme emerging from the women’s experiences was the persistence of the men’s harassment (*n* = 14, 5.23%), wherein men often followed women across different social media accounts or stalked their online activities. The following respondent described her experiences with men across multiple platforms:

There are many strangers who message me on social media, they sent inappropriate messages and pictures, and when I don’t reply on Facebook they begin stalking on other social media accounts. They submit comments on pictures, resulting me to block them [sic].

Related to this, women noted the aggressiveness and escalation of threat with which men responded to their attempts to stop the communication, as the following respondent described:

Some random dude on Instagram started messaging me saying he had videos and photos of me doing sexual things (I’m a virgin), blocked him but he had 4 MORE accounts and insisted that he would sent it to my friends and family.

These reported experiences may reveal why many women feel uncomfortable with direct refusals of male sexual attention. The fact that these women were completely unfamiliar with these men makes their behavior unpredictable and potentially dangerous. The following examples show the reality faced by women who are subjected to unwanted harassment by strange men:

A guy who I had met on a video game and had as a friend on skype constantly wanted to call me and got mad whenever I didn't pick up. He called me roughly 15 times a day and got mad even when I told him I was extremely sick and was napping.

Another participant shared a story of sudden aggression following persistent unwanted sexual communication:

We were chatting and they suddenly suggested we send each other suggestive pictures and even though I said no they were still persistent and sent the pictures anyways and then they told me to send pictures as well and when I said no, they lashed out.

Taken together, these open-ended responses paint a troubling picture in which women are targeted for online sexual harassment beginning at a very young age. This continues throughout adolescence and early adulthood, with many obscene messages and comments being made randomly and without warning. Sometimes the harassment continues across multiple platforms, such that the young woman may feel that there is no escape from the harassing behaviors of men who intrude on their personal online space. Active attempts to shut down the communication may be met with hostility, insults, threats, and aggression. In these ways, the harassment of women online parallels that which occurs in the streets.

## Discussion

Sexual harassment of women occurs not only on streets, sidewalks, bars, and campuses, but has evolved to occupy women’s online spaces as well, finding new ways to harass through social media, smartphones, and online gaming. Prior research has established that women experience a high prevalence of street-level stranger-perpetrated harassment, with 85% in Macmillan et al.’s (2000) study reporting some form of stranger-perpetrated, and over 70% of Fairchild and Rudman’s (2008) sample. The present research shows that women are experiencing comparable rates of male stranger-perpetrated TFSV as well, with approximately half reporting stranger sexual harassment online, most often experiencing multiple forms. These are likely conservative estimates of overall rates of stranger-perpetrated TSFV, given that there are many more websites and platforms that we did not explicitly include in the present study. Additionally, it is possible that some women may not have experienced TFSV on certain platforms depending on their privacy settings. For example, when strict privacy settings are enabled, messages from strangers may be filtered out, which would prevent the user from either receiving or viewing the messages. However, it is important to note that enabling stricter privacy settings can also take away from user experience. For example, on Facebook, privacy settings can prevent anyone who is not a Facebook friend from friending or messaging you. This would also prevent potential real friends, with whom the user would like to engage, from finding them.

It is also important to note that many participants described receiving messages that appeared to be a misguided attempt at obtaining sexual reciprocation. For example, some respondents discussed receiving invitations to exchange explicit photos, or offers of money in exchange for sexual favors. This may also tie into conceptions of sexual entitlement, the societal belief that men are entitled to sex and sexual favors (Bouffard, 2010; Cairns, 1994; Mann & Beech, 2003; Pemberton & Wakeling, 2009). There is also a well-documented tendency for men to overperceive sexual interest in potential partners (Farris et al., 2008; Haselton, 2003). Thus, it is possible that some men are mistakenly seeking sexual opportunities where none exist or that they are playing a game of chance—the more pictures or messages they send out, the more likely at least one woman will reciprocate (Oswald et al., 2019). The low stakes risk posed by sending a message, text, or comment may outweigh the risk of rejection from a relative stranger (Joel et al., 2019; March & Wagstaff, 2017). This perspective, if true, callously ignores the negative impact that men’s unwanted sexual intrusions have on their recipients.

It is noteworthy that in many instances, the perpetrator was not anonymous. Some authors have commented that some forms of TFSV may be analogous to exhibitionism, particularly those in which intimate images and “dick pics” are sent to unsuspecting women (Bader et al., 2008). These men may be more deviance-motivated, seeking excitement and sexual arousal at the thought of women experiencing shock at the sight of their genitalia. These different potential motivations and forms of harassment are suggestive of a potential typology of stranger-perpetrated TFSV. Anonymous stranger-perpetrated TFSV, like “cyberflashing,” may be perpetrated with the intent to shock or surprise, while nonanonymous stranger-perpetrated TFSV like soliciting nude photos or sending explicit messages may be done as a misguided attempt at flirtation or sexual reciprocation based on a presumed entitlement to sex. Other motivations may be based on a sense of “aggrieved entitlement” (Kimmel, 2013, p. 18) in which men express resentment and hostility toward women’s advancement in American society. Further research is warranted to determine whether such a perpetrator typology may in fact exist and what forms it might take.

The majority of our sample were relatively young, and most had only recently graduated high school. Despite this, a very high percentage of our sample reported multiple, even regular experiences with male sexual harassment online. Such prevalent exposure may have important consequences for girls’ developing ideas about their own sexuality, healthy intimate relationships, or the importance of active consent in sexual relationships. It is apparent that, like street-level harassment, online sexual harassment has become almost a rite of passage as young girls mature into adulthood (Saunders et al., 2017). In the same way that women are socialized to play along or brush aside male sexist and harassing comments in bars and streets, women are now socialized to simply ignore or delete online comments and accept that there will always be the potential for “dick pics” and sexually degrading comments to their personal posts. As McGlynn et al. (2017, p. 40) note, “women are still being told to ‘just ignore’ such conduct … to get a sense of humor, to take it easy.” This aligns with social control theories of men’s sexual harassment of women, in which harassment is used to reinforce power inequalities (Fitzgerald, 1993). This is often seen indirectly in the way that women avoid walking alone at night or modify their behavior to avoid sexual assault (Lenton et al., 1999). Similarly, women are forced to modify their actions online through self-censorship or caution to avoid unwanted sexual intrusions on their social media, smartphones, or other technology-based applications.

McGlynn et al. (2017) argue that this regular and unwanted intrusion into women’s lives may be situated within Kelly’s (1988) continuum of sexual violence, which proposes that women’s experiences of sexual harm cannot be and should not be defined solely by legal definitions. Rather, women experience many forms of unwanted sexual intrusion and harm, in both committed consensual relationships as well as from strangers. Sexual harm may take many forms, with the continuum of violence ranging from pressure to coercion and force. The common underlying theme of sexual harm involves the control of victims through “abuse, intimidation, coercion, intrusion, threat and force” (Kelly, 1988, p. 76).

What emerged from the present research was a clear message that these young women did not appreciate or enjoy men’s intrusions into their online space. Collapsing across the various forms of TFSV, the most commonly reported emotional reactions were annoyance, anger, disgust, and fear; the least commonly experienced emotions were lust, excitement, pride, and feeling sexy. Oswald et al. (2019) found that the most common reason men distributed unsolicited “dick pics” was the hope of sexual reciprocity and the most common emotional reaction men anticipated was that the female recipient would feel sexually excited and attractive. The present results confirm that there is a clear disconnect between the goal and the result, evidencing that sending sexual messages, images, comments or propositions online is a poor strategy for sexual reciprocation.

Women reported generally nonconfrontational strategies for dealing with male harassment online, much as they do with street-level harassment (Fairchild & Rudman, 2008). It is not surprising why this may be the case. Women in our study reported that, when they confronted the men actively and directly, some men lashed out at them with aggression and escalation of hostility. Some men were not willing to take no for an answer, and with the availability of online search capacities it is impossible to know whether the male stranger will track down a woman’s address for home or work. This possibility may lead some women to adopt a less adversarial approach, ultimately removing themselves from the harasser’s gaze by deleting her account. A man willing to send unsolicited nude photos of himself, who stalks across platforms and makes explicit threats may be capable of in person harm as well. Because he is a stranger, the woman does not know which threats are empty and which are credible.

### Policy Implications

To address the problem of persistent TFSV, we may look to industry for solutions. Many social media and online platforms such as Facebook, Twitter, and YouTube, have written codes of conduct for acceptable use as well as mechanisms in place to report abuse. The efficacy of these reporting and enforcement mechanisms, however, is questionable, as it often pertains only to credible threats of death or serious harm, or to hate speech (which itself is given quite wide latitude under freedom of expression laws, Bell, 2009). Offensive content could be removed by the end user, the internet service provider or the online platform itself, and repeated violations could result in blocking an account or user. This is a less than satisfactory outcome, as it requires a level of persistence and repeated harm to the victim and does not prevent the offender from opening a new account to continue their harassment. Anecdotally, private individuals have developed their own filters to block unsolicited “dick pics” and explicit content, sharing this filter freely with anyone who wishes to use it (Chu, 2019).

There have been several attempts at creating safeguards against stranger-perpetrated TFSV, specifically against cyberflashing. In 2019, the Bumble application launched a feature named the “Private Detector,” which uses artificial intelligence to detect dick pics in order to blur the image and alert the user. The user can then choose whether to view or block the image, and if they desire, report the image to the moderation team (Moss, 2019). In 2018, in response to the disturbing trend of AirDropping “dick pics” on unsuspecting strangers taking public transit, New York City proposed a bill that would criminalize this conduct in the city. Henry and Powell (2018) note that much remains to be done to create legal remedies for TFSV, and that there are legal complexities around the intentions of the perpetrator (e.g., whether they intended psychological harm), shifting standards of reasonableness, and concepts around the harms caused to the victim. Although criminal sanctions are encouraged as a legal remedy for victims, it is prudent to also consider preventative measures to reduce their occurrence.

The young age at which much of the reported harassment began for participants demonstrates the need to begin intervention strategies in high school or even earlier. At the present stage, there is insufficient research on this topic to make clear recommendations regarding the content or nature of these early intervention strategies, and further research is warranted. Although online sexual harassment may seem more benign than in person harassment given the victim’s ability to delete the offensive content or close their account, this is a faulty assumption. First, much of this sexual exposure occurs to girls at young ages, well before they should be exposed to sexualized content of any sort. Second, the experience of harassment and exposure to graphic content is aversive, offensive, and sexually degrading to its victims. The damage is done whether the photo is deleted or not. Third, it is unreasonable to expect women to continuously monitor their own accounts to avoid male predation, nor is it reasonable to expect women to delete their personal accounts to deal with inappropriate male behavior. Fourth, there are also costs associated with self-monitoring and self-censorship online. Having to maintain strict privacy settings can reduce opportunities for women to connect with friends/acquaintances or engage in professional networking opportunities. Limiting personal expressions online can impair artistic or creative output and self-promotion. Moreover, to the extent that men are not similarly having to hide behind privacy settings or restrict their self-promotion online, gender disparities in employment or professional opportunities are exacerbated.

### Limitations and Future Research

As with all research, the present study is not without limitations. We elected to survey university-aged women as this group constituted the very demographic we suspected to be at the highest risk of TFSV (Henry & Powell, 2018), and our results have demonstrated the high rates at which this group did experience online sexual harassment. Future research should explore harassment experiences of young women outside of a university context, as well as women of older age groups. For this study, we relied on an online format for data collection in the belief that this might elicit greater disclosure of private or embarrassing experiences and provide greater anonymity to participants. We still believe that continued use of online responding would be appropriate for large-scale data collection but acknowledge the value of also employing in person interview and focus groups going forward to obtain rich and in-depth qualitative work.

Follow-up research may wish to include a measure of how frequently participants spend time online as a control variable, as it is intuitive that the more time women spend online, the greater their potential exposure to harassment. This may also include a measure of which sites women are using most often (e.g., Facebook, Instagram) to understand where the risk of harassment is greatest. Based on a preliminary assessment of our data, it appears that Instagram, Snapchat and Facebook are prime targets for inappropriate messages and photos. Additional measures may probe for more nuanced emotional and behavioral reactions to online sexual harassment. In the present study, we assessed behavioral and emotional reactions to any form of TFSV, but it is possible that different forms will result in different psychological consequences for women. For example, there is some recent research documenting that women’s reactions to receiving unsolicited “dick pics” involves shame, disgust, anger, objectification, and feelings of harassment (Vitis & Gilmour, 2017; Waling & Pym, 2019). Less is known about how women might react to other forms of male online sexual harassment, and future research should explore this in more focused detail.

In this study, we used a liberal definition of the word stranger. This was done for logical purposes, acknowledging that online-perpetrated stranger harassment is markedly different than in-person, and not always perpetrated by an unknown passerby on the street. We placed several safeguards in the study to ensure that respondents understood what we meant by stranger, including an assessment at the beginning that did not allow respondents to continue in the survey unless they responded correctly to a question probing them about what a “stranger” was according to our study. However, regardless of the safeguards, it is possible that this definition could have been interpreted differently by respondents.

We allowed participants to select categories that best reflected their experiences. It is possible that the same experience could have been captured by multiple categories, which makes the absolute prevalence figures of each experience less clear. However, we were more interested in how these actions were interpreted and experienced from the perspective of the victim. Additionally, as previously stated, this research was largely exploratory in nature. Future research should focus on better understanding how prevalent these experiences are in women’s day-to-day online experiences.

Baptist and Coburn (2019) argue that we must consider intersectional identities when discussing stranger harassment, noting that nonheterosexual women and women of color face a disproportionate amount of stranger-perpetrated harassment at street level. Vera-Gray (2016) notes that the intersection of race, class, appearance, and sexual orientation all mutually inform whether a woman will interpret cat calling as harassing or intimidating. Moreover, she notes that women of color are more likely to be subjected to sexually explicit speech and racially motivated comments than are White women. The present research was not designed to address this complex issue fully, nor did we have a sufficient number of participants representative of varying ethnic identities or sexual orientations. Future research should explore how these intersectional identities shape a woman’s experiences with male harassment in an online context.

Finally, we recommend that future directions consider the prevalence of stranger sexual harassment against men, both female-perpetrated and same-sex harassment. Little is known about how often men may be subjected to similar forms of stranger-perpetrated TFSV, the forms it might take, and its consequences.

## Conclusions

The present research provides a novel exploration into the experiences of young women with stranger sexual harassment in an online context. The present research has shown that women generally find the experience of male sexual harassment online to be unwanted and unpleasant, with many downstream negative consequences. Although women who experience street-level harassment self-isolate and remain off the streets, women experiencing online sexual harassment have engaged in similar actions by removing their accounts, hiding their profiles, or avoiding posting photographs of themselves. The high prevalence of sexual harassment of these young women, most having only recently graduated from high school, provides cause for concern. Based on our analysis of their open-ended comments and self-reported emotional reactions, these women interpreted male sexual attention in an aversive and unpleasant manner. It is generally not the case that women enjoyed the attention or felt merely impartial to it; quite the contrary. Clearly, the growing phenomenon of TFSV is a pervasive and harmful way that some men have found to intrude upon women’s space. Intervention strategies that target both young men and young women about safe and respectful online behavior are clearly warranted, as well as more meaningful sanctions for violations of online terms of use agreements.

## Author Biographies

**Alisha C. Salerno-Ferraro,** MA, is a PhD candidate at York University. Her research focuses on police response to autism spectrum disorder, technology-facilitated sexual violence, and legal decision-making. She employs both quantitative and qualitative research methods to produce evidence-based publications to help inform policy makers.

**Caroline Erentzen,** PhD, is a postdoctoral fellow at the University of Toronto. Her research focuses on the gendered dimensions of Islamophobia and the ways that gender, age, and religion intersect to predict stereotyping and confrontations of prejudice.

**Regina A. Schuller,** PhD, is a professor of psychology at York University. Her research explores the intersection of psychology and law, including intimate partner violence, sexual violence, wrongful convictions, and jury racial bias.
